# Left upper division segmentectomy with a simultaneous displaced bronchus and pulmonary arteriovenous anomalies: a case report

**DOI:** 10.1186/s13019-018-0741-6

**Published:** 2018-05-16

**Authors:** Kazuki Hayashi, Makoto Motoishi, Kanna Horimoto, Satoru Sawai, Jun Hanaoka

**Affiliations:** 1 Division of General Thoracic Surgery, Department of Surgery, Shiga University of Medical Science, Setatsukinowa-cho, Otsu, Shiga 520-2192 Japan; 2 Department of Thoracic Surgery, Mitsubishikyoto Hospital, 1 Katsuragosyo-cho, Nishikyo-ku, Kyoto 615-8087 Japan; 3 Department of Thoracic Surgery, National Hospital Organization Kyoto Medical Center, Fukakusamukaihata-cho, Fushimi-ku, Kyoto 612-8555 Japan

**Keywords:** Lung cancer, Displaced bronchus, Pulmonary artery anomaly, Pulmonary venous anomaly, Accessory fissure

## Abstract

**Background:**

A displaced bronchus is a rare disorder of the left upper lobe. Displaced bronchi are often accompanied by an anomaly of a pulmonary artery, but rarely of a pulmonary vein.

**Case presentation:**

We here present a patient with primary lung cancer and simultaneous migration abnormalities of the pulmonary artery and vein in a displaced bronchus of the left upper lobe. Previous reports and our findings indicate that anomalies of the pulmonary artery and vein combined with a displaced bronchus of the left upper lobe have the following characteristics: (1) the left main pulmonary artery does not cross the dorsal side of the displaced bronchus; (2) V^1 + 2^ returns to the inferior pulmonary vein; and (3) there is an accessory fissure (aberrant fissure) in the segments dominated by the displaced bronchus.

**Conclusions:**

Prevention of intraoperative damage during procedures for a displaced bronchus and pulmonary arteriovenous anomalies requires careful preoperative evaluation and surgical technique with particular attention to the above-listed characteristics.

## Background

Many bronchial bifurcation abnormalities have been reported in the right upper lobe [1–3]; however, such abnormalities are rare in the left upper lobe. A displaced bronchus is frequently accompanied by the pulmonary artery taking an abnormal course [4–15], but few patients in whom the pulmonary vein also takes an abnormal course have been reported. We here report performing a segmentectomy for a left upper lobe lung cancer in a patient with a displaced bronchus in whom the left main pulmonary artery and superior pulmonary vein both took abnormal courses.

## Case presentation

A 78-year-old man was referred to our department for suspected left upper lobe lung cancer (cT1cN0M0 stage IA3). He had a history of percutaneous coronary intervention for angina pectoris, and internal carotid artery stenosis and hypertension for which he was receiving medical treatment. He had been a heavy smoker. The Brinkman Index was 1000. On chest auscultation, fine crackles were heard on the back bilaterally. Blood tests showed no abnormal findings other than a high carcinoembryonic antigen concentration (7.2 ng/mL). Pulmonary function tests showed an obstructive disorder as evidenced by a forced expiratory volume 1.0% (FEV 1.0%) (G) of 68.8%.

**Fig. 1:**
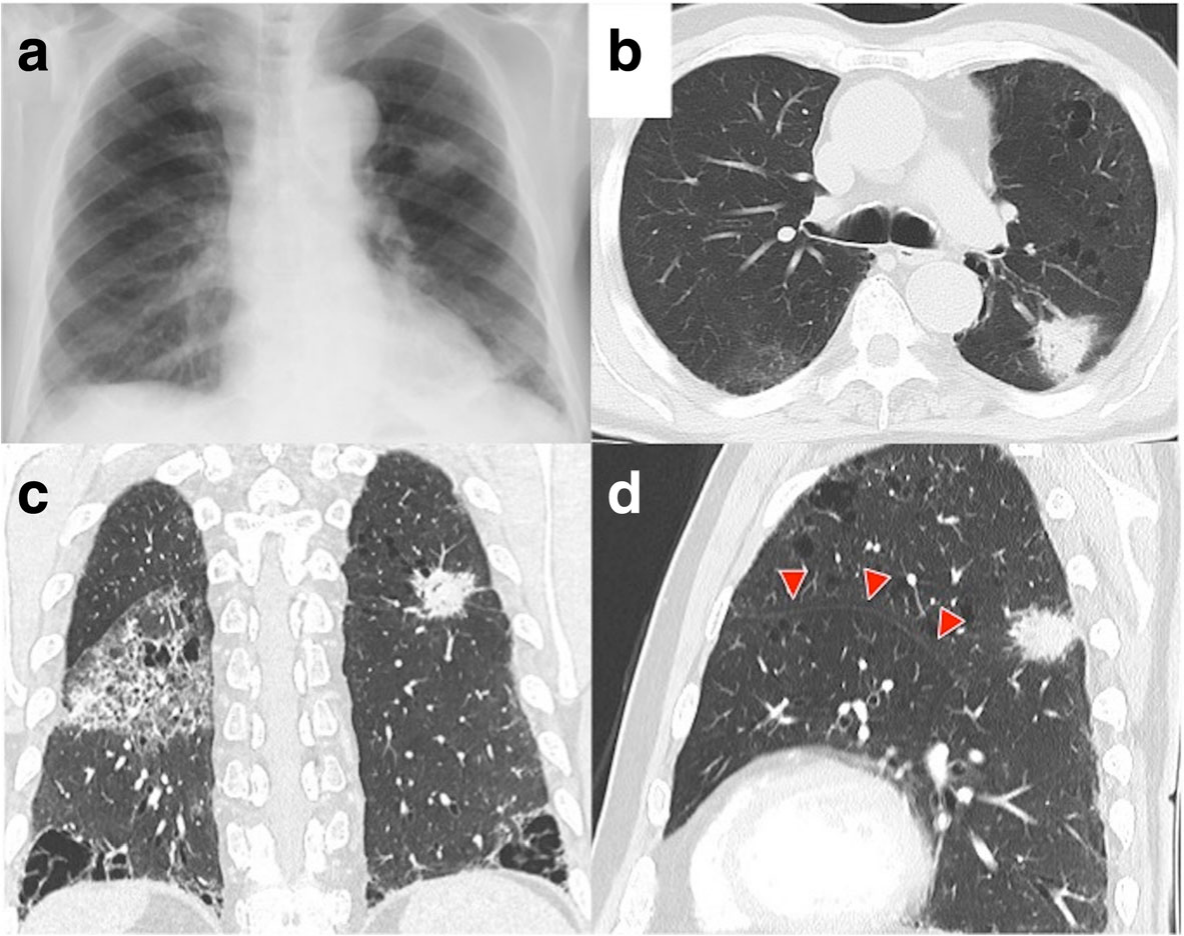
**a**, **b** Chest radiograph and CT image showing a mass in the left lung S^1 + 2^ segment. **c** Chest CT image showing a mass, emphysema in the upper lobes, and fibrosis in the lower lobes. **d** Chest CT image showing an accessory fissure between S^1–3^ and S^4 + 5^ (arrowheads)

A chest radiograph and chest computed tomography (CT) showed a tumor of maximum diameter 30 mm in the left lung S^1 + 2^ segment (Fig. 1a, b), emphysema of the upper lobe, and fibrosis of the left lower lobe. The patient was diagnosed as having combined pulmonary fibrosis and emphysema (Fig. 1c). Additionally, an accessory fissure (aberrant fissure) was identified between the S^1–3^ and S^4 + 5^ segments (Fig. 1d). Bronchoscopy had been performed at the referring institution and was not repeated in our department. Bronchoscopic images were not viewed preoperatively and no bronchial abnormalities were mentioned in the report of the referring institution’s bronchoscopy. No histological diagnosis had been obtained by bronchoscopy.

**Fig. 2:**
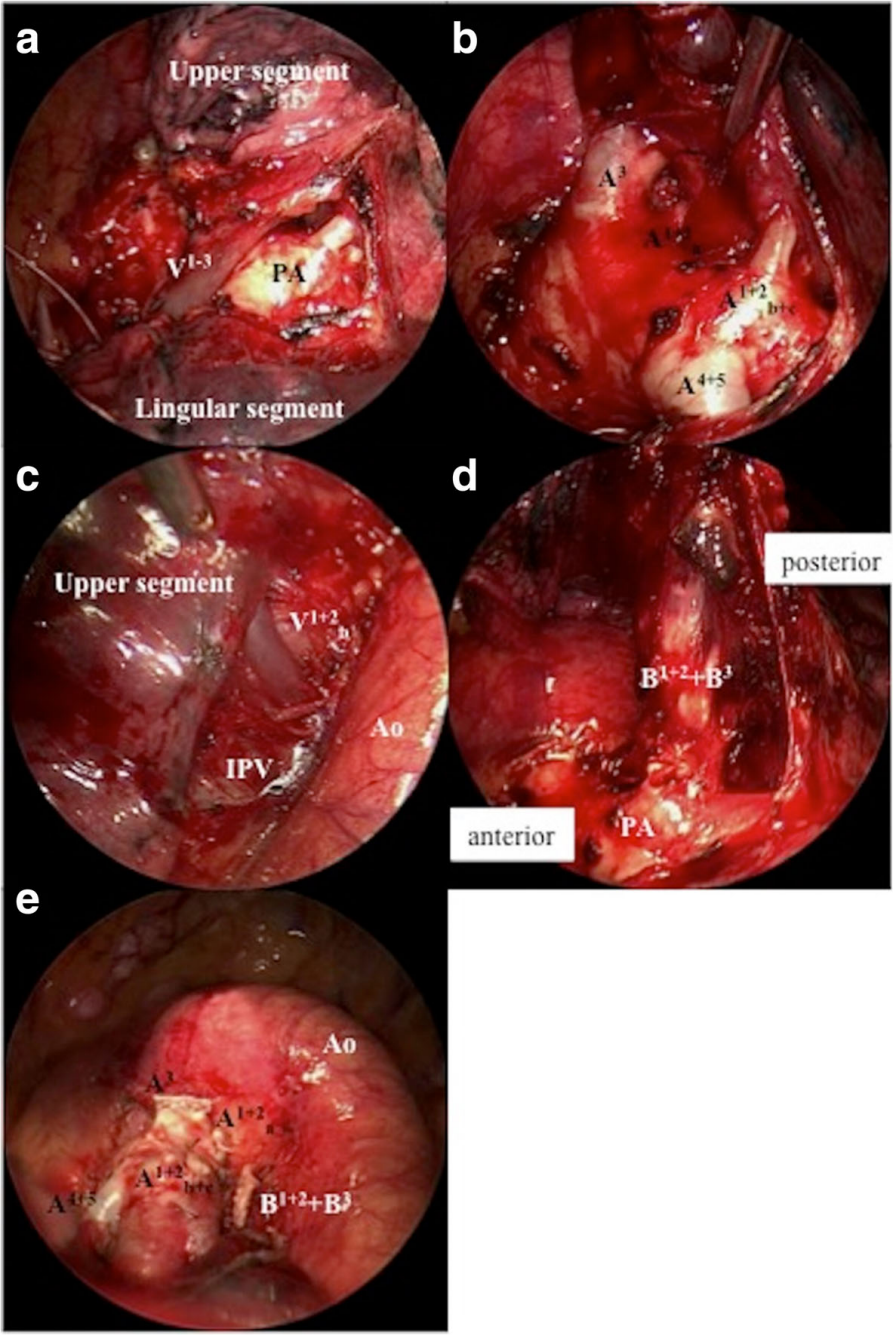
**a** Intraoperative image showing V^1–3^ on the ventral side of the mediastinum. **b** Intraoperative image showing the branches of A3, A^1 + 2^_a_, A^1 + 2^_b + c_, and A^4 + 5^. **c** Intraoperative image showing V^1 + 2^_b_ joining the IPV. (D) Intraoperative image showing B^1 + 2^ + B^3^ on the dorsal side of the left main pulmonary artery. **e** Intraoperative image after left upper division segmentectomy. IPV, inferior pulmonary vein

In consideration of the patient’s age, his combined pulmonary fibrosis and emphysema, and the presence of an accessory fissure between S^1–3^ and S^4 + 5^, left upper division segmentectomy by video-assisted thoracoscopic surgery was selected as the procedure of choice. V^1–3^ was identified on the ventral side of the mediastinum and dissected (Fig. 2a). The branches of A^3^, A^1 + 2^_a_, A^1 + 2^_b + c_ were identified and divided (Fig. 2b) by reaching the pulmonary arterial wall from the accessory fissure of S^1–3^ and S^4 + 5^. Subsequently, the IPV and V^1 + 2^_b_, which joined the IPV, were identified on the dorsal side of the mediastinum, and V^1 + 2^_b_ was divided (Fig. 2c). B^1 + 2^ + B^3^, which were on the dorsal side of the left main pulmonary artery, were identified and divided (Fig. 2d). Incomplete lobulation was noted dorsally between the upper and lower lobes. Therefore, to secure sufficient tumor margins, part of S^6^ was included in the upper lobe and the incomplete lobulation was divided with a stapler. S^1–3^ was then excised (Fig. 2e). The operation time was 4 h and 49 min and amount of bleeding was 30 mL.

**Fig. 3:**
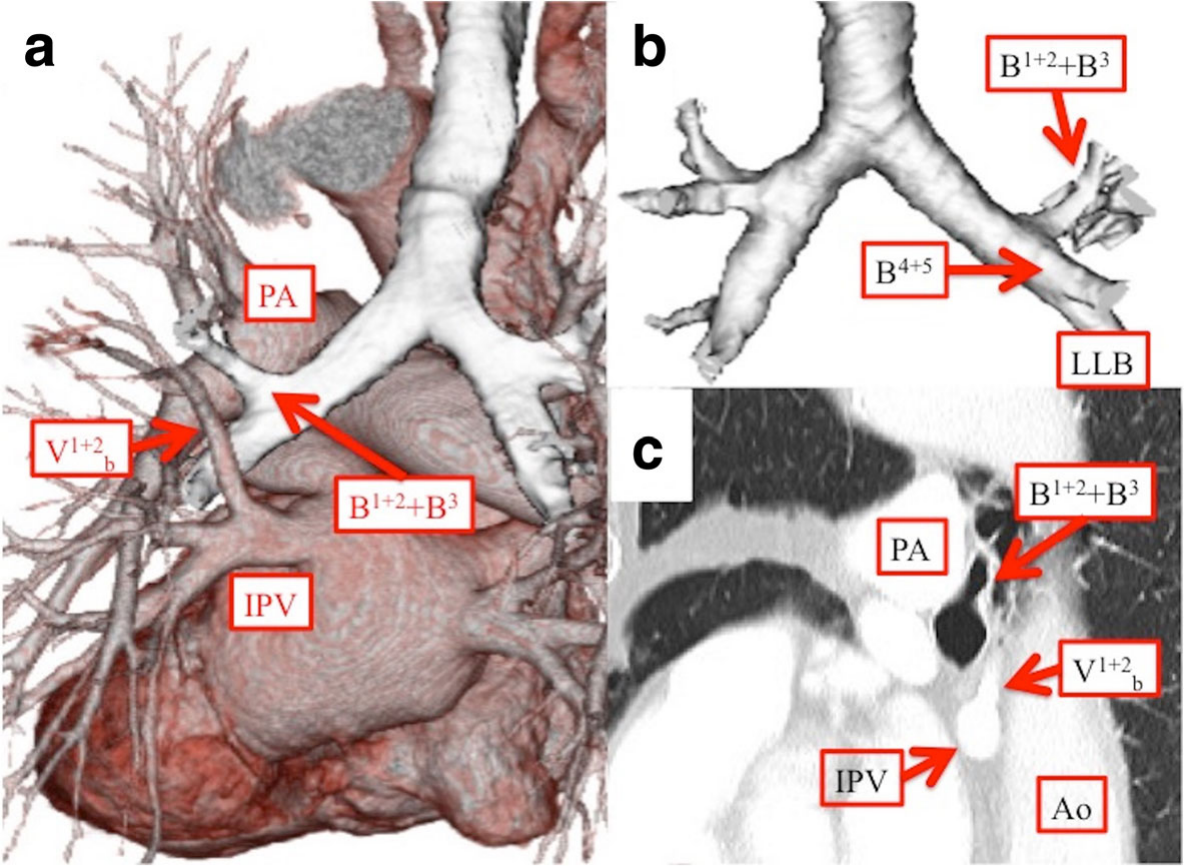
**a**, **c** Three-dimensional reconstructed image and chest CT image (sagittal) showing that the left main pulmonary artery is on the ventral side of B^1 + 2^ + B^3^ and does not cross the dorsal side of the left main bronchus and that V^1 + 2^_b_ joins the IPV on the dorsal side of the pulmonary artery. **b** Three-dimensional reconstructed image showing that B^1 + 2^ + B^3^ branches from the left main bronchus, followed by B^4 + 5^ and the lower lobe bronchus. Ao, aorta; IPV, inferior pulmonary vein; LLB, left lower bronchus; PA, pulmonary artery

Three-dimensional reconstructed images of the pulmonary artery and vein had been prepared preoperatively, whereas images that also included the bronchi were only prepared postoperatively. The latter showed the following: B^1 + 2^ + B^3^ was branching from the left main bronchus, followed by a bronchus that corresponded to the middle bronchial trunk of the right lung, and B^4 + 5^ and the lower lobe bronchus (Fig. 3a, b); the left main pulmonary artery was on the ventral side of B^1 + 2^ + B^3^ and did not cross the dorsal side of the left main bronchus; and V^1 + 2^_b_ joined the inferior pulmonary vein (IPV) on the dorsal side of the pulmonary artery (Fig. 3a). In addition, when the image of the sagittal section was reviewed again postoperatively, the above listed features were visible on plain CT (Fig. 3c).

**Fig. 4:**
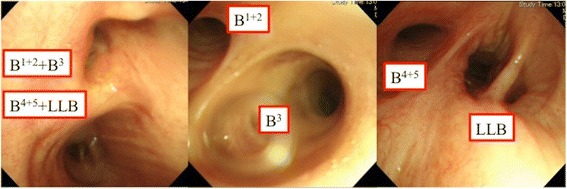
Bronchoscopy showing B^1 + 2^ + B^3^ branches from the left main bronchus, followed by B^4 + 5^ and the lower lobe bronchus

Additionally, postoperative review of available bronchoscopic images revealed an abnormal bronchial course (Fig. 4).

Pathological examination of the resected specimen resulted in a diagnosis of adenocarcinoma with metastases in the mediastinal lymph nodes and of pathological stage pT1cN2M0 stage IIIA. The postoperative course was uneventful and the patient was discharged on postoperative day 8. No recurrence had occurred by 1 year after surgery.

## Discussion

A displaced bronchus is defined as one that branches away from its customary position [16]. The prevalence of this anomaly is only 0.64 and 75% are in the right upper lobe [17]. A displaced bronchus in the left upper lobe is rare, and one with pulmonary arterial and venous abnormalities even rarer. To the best of our knowledge, 15 surgical cases of a displaced bronchus in the left upper lobe have been reported, including the present one [4–16, 18, 19]. Of these, only four patients, including our patient, had pulmonary arterial and venous malformations (Table 1). About 75% of pulmonary veins with an abnormal course involve V^1 + 2^. Features that are common to anomalies of the pulmonary artery and vein combined with a displaced bronchus of the left upper lobe are as follows: (1) the left main pulmonary artery does not cross the dorsal side of the displaced bronchus; (2) V^1 + 2^ returns to the inferior pulmonary vein; and (3) there is an accessory fissure in the segments dominated by the displaced bronchus.

**Table 1:** Previous reports of lung resection in patients with a displaced bronchus of the left upper lobe

First author	Year	Age (y)/sex	Diagnosis	Displaced bronchus	Position of displaced bronchus	Lobulation	Procedure	Anomalous PV
Anno	1959	28/M	Tuberculosis	B^1 + 2^ + B^3^/B^4 + 5^	Behind the MPA	S^4 + 5^/S^1 + 2 + 6–10^	S^1–3^ Seg.	V^1 + 2^
Takahashi	1992	47/F	Sclerosing hemangioma	B^1 + 2^/B^3^ + B^4 + 5^	N.D.	S^3–5^/S^1 + 2 + 6–10^	S^3–5^ Seg.	N.D.
Motohashi	1995	52/F	Lung cancer	B^1 + 2^_a_/B^1 + 2^_b,c_ + B^3^/B^4 + 5^	N.D.	N.D.	Pneumonectomy	None
Okamoto	1999	59/M	Lung cancer	B^1 + 2^ + B^3^/B^4 + 5^	Behind the MPA	S^1–3^/S^4–10^	LLL	None
Shimamoto	2008	81/F	Lung cancer	B^1 + 2^/B^3^ + B^4 + 5^	Behind the MPA	S^1–5^/S^6–10^	S^1 + 2^ Seg.	N.D.
Tarukawa	2010	65/M	Lung cancer	B^1 + 2^ + B^3^/B^4 + 5^	Behind the MPA	S^1–3^/S^4–10^	S^1–3^ Seg.	N.D.
Tsukioka	2011	62/F	Lung cancer	B^1 + 2^/B^3^ + B^4 + 5^	Behind the MPA	S^1–2^/S^3–10^	LUL	N.D.
Ikuta	2013	83/M	Lung cancer	B^1 + 2^/B^3^ + B^4 + 5^	Behind the MPA	S^1–2^/S^3–10^	LUL	V^1 + 2^
Katayama	2013	55/M	Lung cancer	B^1 + 2^ + B^3^/B^4 + 5^	Behind the MPA	S^1–3^/S^4+ 5^/S^6–10^	S^1–3^ Seg.	N.D.
Asakura	2014	52/M	Lung cancer	B^1 + 2^/B^3^ + B^4 + 5^	Behind the MPA	S^1–2^/S^3–10^	LUL	N.D.
Kawano	2014	59/M	Lung cancer	B^1 + 2^ + B^3^/B^4 + 5^	Behind the MPA	S^1–3^/S^4–10^	S^1–3^ Seg.	None
Yaginuma	2015	63/F	Lung cancer	B^1 + 2^ + B^3^/B^4 + 5^	Behind the MPA	S^1–3^/S^4 + 5^/S^6–10^	S^1–3^ Seg.	N.D.
Chiba	2015	57/M	Lung cancer	B^1 + 2^ + B^3^/B^4 + 5^	Behind the MPA	S^1–3^/S^4 + 5^/S^6–10^	S^1–3^ Seg.	Common duct of the SPV and IPV
Onuki	2016	83/F	Lung cancer	B^1 + 2^/B^3^ + B^4 + 5^	Behind the MPA	S^1 + 2^/S^3^/S^4+ 5^/S^6–10^	LUL	None
Present case	2016	78/M	Lung cancer	B^1 + 2^ + B^3^/B^4 + 5^	Behind the MPA	S^1–3 + 6^/S^4+ 5^/S^8–10^	S^1–3^ Seg.	V^1 + 2^

*M* male, *F* female, *N.D.* not described, *LUB* left upper lobe bronchus, *MPA* main pulmonary artery, *Seg*. segmentectomy, *LLL* left lower lobectomy, *LUL* left upper lobectomy, *SPV* superior pulmonary vein, *IPV* inferior pulmonary vein

Surgeons need to pay attention to these features when performing preoperative evaluation and surgery. In our case, that the pulmonary vein had an abnormal course was identified by preoperative CT, whereas the abnormal courses of the bronchus and pulmonary artery were first recognized intraoperatively. Preoperative images should be carefully evaluated; however, details of any abnormal anatomy may be difficult to identify or assess on axial images. Additionally, three-dimensional reconstructed images including the bronchi are not always prepared preoperatively. Therefore, how to adequately evaluate a surgical candidate preoperatively without missing important features is an issue that needs to be addressed. One means of addressing this issue could be to focus on accessory fissures. Because accessory fissures are the most easily detected of the above-mentioned abnormalities, whenever an accessory fissure is recognized the surgeon must investigate the possibility of blood vessels or bronchi having abnormal courses. An accessory fissure and abnormal courses of bronchi and blood vessels are often present simultaneously [20]; the reason for this association is unclear.

Nine of the 15 reported patients with a displaced bronchus in the left upper lobe had accompanying incomplete lobulation between the upper and lower lobes [4, 5, 7–9, 11, 12, 18]. There is a tendency for incomplete lobulation between the upper and lower lobe when there is an accessory fissure in the upper lobe. In such cases, this incomplete lobulation needs to be divided during lung resection. However, in some previously reported patients, the abnormal courses of blood vessels and the bronchus had not been detected preoperatively; thus, some of them were damaged during division of incomplete lobulation [6, 8]. In particular, with a pulmonary venous anomaly such as our patient had, V^1 + 2^ lies to the dorsal side of the mediastinum, making it highly likely that it will be damaged when dividing incomplete lobulation. The no-touch fissure technique is useful for preventing intraoperative damage when performing lobectomy or segmentectomy in a patient with a displaced bronchus, [11]. This technique requires dividing of the pulmonary artery and bronchus before dividing incomplete lobulation. However, even when using a technique that involves dividing blood vessels and bronchi first, there is still a danger of intraoperative damage if the surgeon has insufficient information concerning the course of blood vessels and bronchi. Patients with anomalies similar to those of our patient tend to have an accessory fissure. In such cases, approaching the pulmonary artery from the accessory fissure and deliberately detaching the blood vessels and bronchus are essential for preventing intraoperative damage.

## Conclusions

When the bronchi and pulmonary artery and veins all take abnormal courses in the left lung, the following is usually present. (1) The left main pulmonary artery does not cross the dorsal side of the displaced bronchus. (2) V^1 + 2^ returns to the inferior pulmonary vein. (3) There is an accessory fissure in the segments dominated by the displaced bronchus. Surgeons need to be aware of these features to prevent damage during surgery. In particular, focusing on any accessory fissure when performing preoperative evaluations may maximize detection of associated anomalies.

## Abbreviations

Ao: Aorta
CT: Computed tomography
FEV 1.0%: Forced expiratory volume 1.0%
IPV: Inferior pulmonary vein
LLB: Left lower bronchus
LLL: Left lower lobectomy
LUB: Left upper lobe bronchus
LUL: Left upper lobectomy
MPA: Main pulmonary artery
N.D.: Not described
PA: Pulmonary artery
Seg.: Segmentectomy
SPV: Superior pulmonary vein
